# Supplementary material for: Genome-wide approach identifies a novel gene-maternal pre-pregnancy BMI interaction on preterm birth
Source: Nat Commun. 2017 Jun 9;8:15608. doi: 10.1038/ncomms15608 (PMC5472707; doi:10.1038/ncomms15608)
Supplement: Supplementary Information — Supplementary Figures and Supplementary Tables [file ncomms15608-s1.pdf]

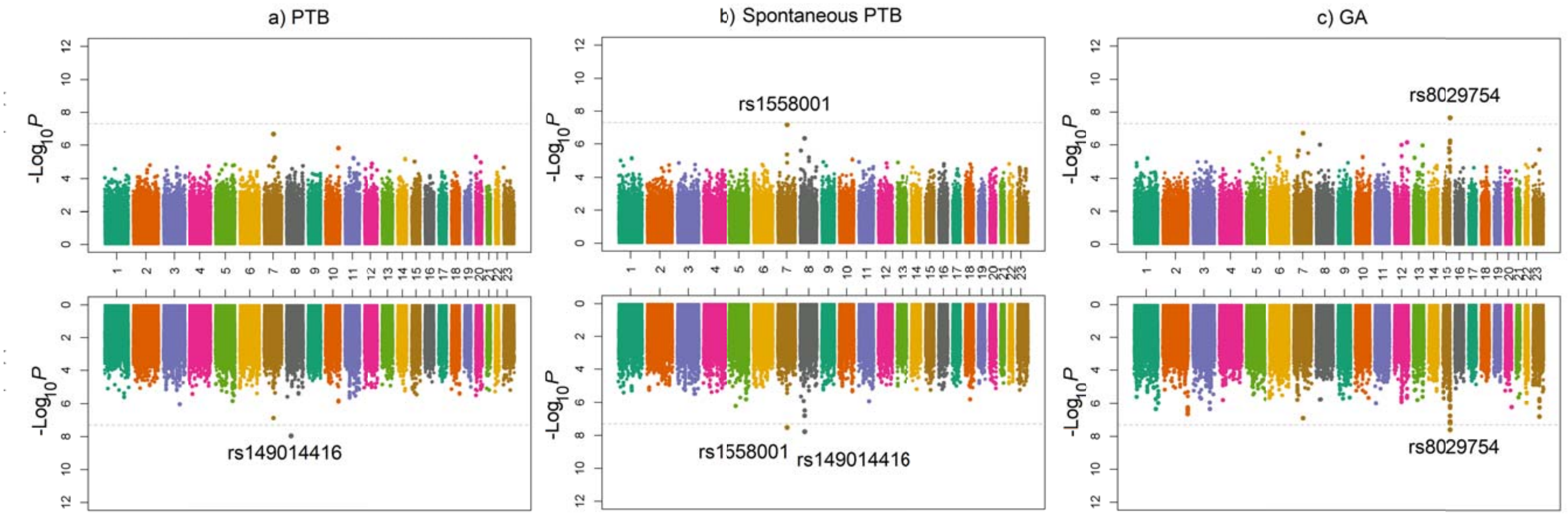

Supplementary Figure 1. Manhattan plots for genome-wide associations of genotyped SNPs and genotyped plus imputed SNPs with PTB outcomes in 1,733 African-American mothers from the Boston Birth Cohort, with adjustment for genotyping batch, genetic ancestry, maternal age at delivery, parity and infant's gender. Fig 1a, 1b and 1c are Manhattan plots for overall PTB, spontaneous PTB, and gestational age at delivery (GA), respectively. In each plot, the upper panel and the lower panel represent the genotyped SNPs and the genotyped plus imputed SNPs, respectively. The dashed line represents the genome-wide significance level with $p=5\times10^{-8}$.

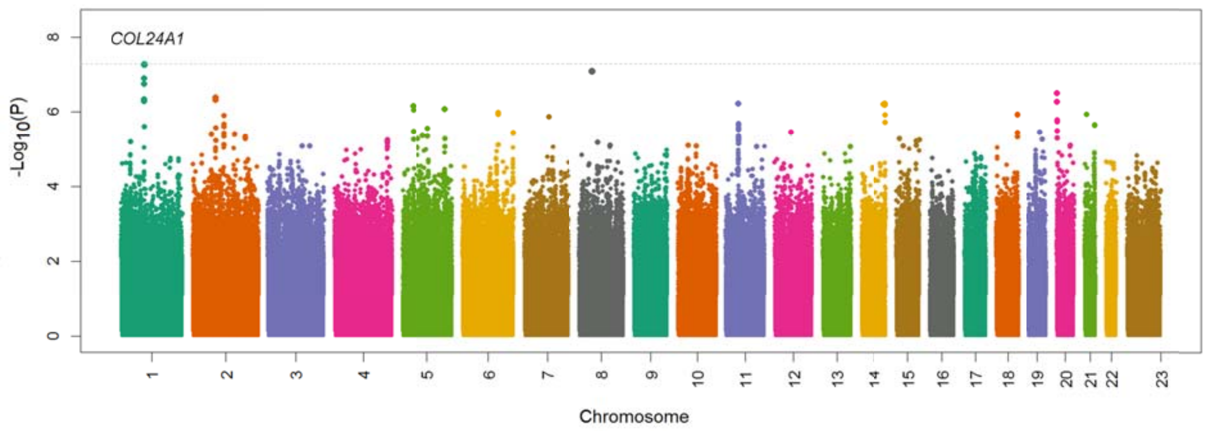

Supplementary Figure 2. The Manhattan plot for genome-wide associations by the 2 degree-of-freedom approach to combine signals of SNP main effects and interaction effects with pre-pregnancy BMI category on overall PTB in 1,586 African-American mothers from the Boston Birth Cohort, adjusted for genotyping batch, genetic ancestry, maternal age at delivery, parity, and infant's gender.

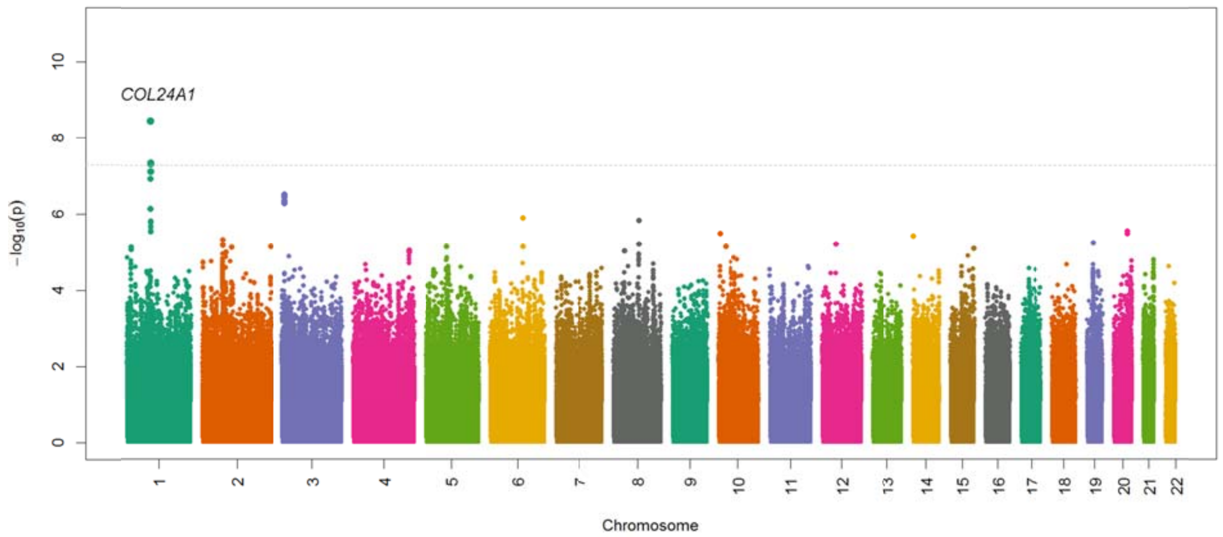

Supplementary Figure 3. The Manhattan plot for genome-wide meta-analyses of SNP× pre-pregnancy BMI interaction on overall PTB in 1,886 African-American mothers from the Boston Birth Cohort and from the GPN study.

Supplementary Table 1. The population characteristics of the two replication cohorts

Variable	GENEVA[a] African American		GPN[a] African American		GPN[b] Caucasian	
	**TB**	**PTB**	**TB**	**PTB**	**TB**	**PTB**
**N**	306	130	169	177	365	318
**Maternal age (year), Mean±SD**	25.7 ± 5.8	26.4 ± 6.3	23.9 ± 5.4	24.4 ± 5.8	25.9 ± 5.5	25.6 ± 5.5
**Pre-pregnancy BMI (kg m^{-2})**						
< 18.5	NA	NA	10 (5.9)	10 (5.7)	0	0
18.5-24.9	NA	NA	67 (39.6)	61 (34.5)	247(67.7)	192 (60.4)
25-29.9	NA	NA	33 (19.5)	42 (23.7)	74 (20.3)	73 (23.0)
≥ 30	NA	NA	49 (29.0)	48 (27.1)	44 (12.0)	53 (16.6)
Unknown	306	130	10 (5.9)	16 (9.0)	0	0
**Infant's gender:** Male	149 (48.7)	65 (50.0)	98 (58.0)	89 (50.3)	186 (51.0)	181 (56.9)
**Nulliparity**	NA	NA	67 (39.6)	70 (39.5)	197 (54.0)	172 (54.1)

TB: Term birth; PTB: preterm birth; SD: standard deviation; BMI: body mass index; NA: not available.

[a] The replication samples are African American mothers from the GWAS of Prematurity and Its Complication (African Americans) (the GENEVA study, dbGaP entry # phs000353.v1.p1), and from the NICHD Genomic and Proteomic Network for Preterm Birth Research (the GPN study, dbGaP entry # phs000714.v1.p1), respectively.

[b] These Caucasian mothers were from the GPN study, all with pre-pregnancy BMI > 18.5 kg m^{-2}.

Supplementary Table 2. Heritability estimates of PTB outcomes and pre-pregnancy BMI in 1,733 unrelated African-American women using genome-wide SNP data[a]

Phenotype	V_G (SE)[b]	Vp (SE)[c]	h^2 (SE)[d]	Transformed h^2 (SE)[e]	*P*
Overall PTB	0.05 (0.05)	0.24 (0.01)	0.21 (0.22)	0.25 (0.27)	0.18
Spontaneous PTB	0.05 (0.05)	0.21 (0.01)	0.22 (0.25)	0.26 (0.29)	0.23
BMI	21.5 (11.4)	43.3 (1.5)	0.50 (0.26)	--	0.03
Height	17.0 (10.7)	46.6 (1.6)	0.37 (0.23)	--	0.002

PTB: Preterm birth; BMI: Body mass index; SE: standard error
[a] Genotyped SNPs with minor allele frequency (MAF) > 0.02 were included.
[b]V_G: The variance explained by genome-wide SNPs with MAF > 0.02
[c]Vp: The total phenotypic variance
[d]h^2: Estimate of genetic variance (V_G) proportional to the total phenotypic variance (Vp) on the observed scale, using the GCTA software.
[e]Transformed h^2: genetic variance proportional to the total phenotypic variance on the liability scale under the assumption that population prevalence is 12% and 8% for overall PTB and spontaneous PTB, respectively.

Supplementary Table 3a. The genome-wide significant loci ($p < 5.0\times10^{-8}$) associated with PTB outcomes in 1,733 African-American women from the Boston Birth Cohort

SNP	CHR	Position	Gene	Allele[b]	MAF[c]	Overall PTB		Spontaneous PTB		GA	
						OR (95%CI)[d]	P[d]	OR (95%CI)[d]	P[d]	Beta±Se[d]	P[d]
rs1558001	7	81407080	*HGF* ǁ *CACNA2D1*	C/T	0.21	1.5 (1.3-1.8)	1.3×10^{-7}	1.7 (1.4-2.0)	3.0×10^{-8}	-0.81±0.15	9.8×10^{-8}
rs149014416[a]	8	*29669769*	*DUSP4* ǁ *LOC101929470*	AG/A	0.03	2.3 (1.7-3.2)	1.1×10^{-8}	2.4 (1.7-3.5)	1.7×10^{-8}	-0.42±0.09	1.8×10^{-6}
rs8029754	15	99979159	*LRRC28* ǁ *MEF2A*	A/G	0.21	1.4 (1.2-1.6)	9.7×10^{-5}	1.5 (1.2-1.7)	7.0×10^{-5}	-0.87±0.15	1.9×10^{-8}

MAF: minor allele frequency; PTB: preterm birth; GA: gestational age. OR: odds ratio; CI: confidence interval.
[a]Imputation score info: 0.98.
[b]Major/minor allele.
[c]Minor allele frequency (MAF) calculated in TB controls.
[d]For each SNP, the allelic dosage of the minor allele was analyzed for its association with each outcome using the frequentist association tests included in SNPTEST, with adjustment for genotyping batch, genetic ancestry, maternal age at delivery, parity, and infant's gender.

Supplementary Table 3b. Replication of the identified genome-wide significant SNPs for their associations with overall PTB in African-American mothers from the two replication cohorts.

SNP[a]	CHR	Position [b]	Allele	The GENEVA study African American			The GPN study African American		
				MAF[c]	OR (95%CI) [d]	P	MAF[c]	OR (95%CI)[d]	P
rs1558001	7	81407080	C/T	0.31	0.7 (0.5-1.0)	0.07	0.22	0.8 (0.6-1.2)	0.37
rs8029754	15	99979159	A/G	0.22	1.0 (0.70-1.42)	0.99	0.21	0.8 (0.5-1.1)	0.19

SNP: single nucleotide polymorphism; CHR: chromosome; MAF; minor allele frequency; OR: odds ratio; CI: conference interval.
[a] The imputed SNP rs149014416 was not available in the two replication samples.
[b] Major/minor allele
[c] Minor allele frequency (MAF) calculated in TB controls
[d] The association between each SNP (additive genetic model) and PTB was analyzed using the logistic regression model, with adjustment for genetic ancestry, parity (only in the GPN study) and infant's gender.

Supplementary Table 4. Consistency of the rs11161721 × pre-pregnancy BMI interaction effect across different PTB subtypes in 1,586 African American mothers from the Boston Birth Cohort

**rs11161721**	**N (%PTB)**			**pre-pregnancy BMI category-PTB association** [a]		
	**NW**	**OW**	**OB**	**OR**	**95%CI**	***P*-value**
**Early PTB (<32 weeks of gestation)**						
CC	323 (9.3)	209 (19.6)	161 (21.1)	1.6	1.2-2.1	7.3×10^{-4}
CA	164 (16.5)	99 (21.2)	105 (11.4)	0.9	0.6-1.3	0.44
AA	19 (10.5)	19 (10.5)	15 (0.0)	0.7	0.1-5.1	0.70
Interaction test [b]						
G×E				0.6	0.4-0.8	0.003
**Late PTB (32 to 36 and 6/7 weeks of gestation)**						
CC	399 (26.6)	267 (37.1)	210 (39.5)	1.4	1.2-1.7	4.3×10^{-4}
CA	217 (36.9)	119 (34.5)	122 (23.8)	0.7	0.6-1.0	0.02
AA	40 (57.5)	24 (29.2)	19 (21.1)	0.4	0.2-0.8	0.01
Interaction test [b]						
G×E				0.5	0.4-0.7	1.3×10^{-7}
**Spontaneous PTB**						
CC	391 (25.1)	249 (32.5)	188 (32.4)	1.2	1.0-1.5	0.03
CA	214 (36.0)	129 (39.5)	116 (19.8)	0.7	0.6-0.9	0.02
AA	35 (51.4)	23 (26.1)	17 (11.8)	0.3	0.1-0.8	0.009
Interaction test [b]						
G×E				0.6	0.4-0.7	1.2×10^{-5}
**Medically-indicated PTB**						
CC	331 (11.5)	227 (26.0)	183 (30.6)	1.9	1.5-2.4	1.8×10^{-7}
CA	167 (18.0)	89 (12.4)	111 (16.2)	0.9	0.6-1.2	0.41
AA	24 (29.2)	20 (15.0)	17 (11.8)	0.6	0.2-1.9	0.42
Interaction test [b]						
G×E				0.5	0.3-0.7	1.7×10^{-5}
**PTB with IUI**						
CC	324 (9.6)	205 (18.0)	162 (21.6)	1.6	1.2-2.1	6.2×10^{-4}
CA	165 (17.0)	99 (21.2)	101 (7.9)	0.7	0.5-1.1	0.09
AA	23 (26.1)	19 (10.5)	16 (6.3)	0.5	0.1-1.8	0.27
Interaction test [b]						
G×E				0.5	0.3-0.7	3.0×10^{-4}
**PTB without IUI**						
CC	398 (26.4)	271 (38.0)	209 (39.2)	1.4	1.1-1.7	5.8×10^{-4}
CA	216 (36.6)	119 (34.4)	126 (26.2)	0.8	0.6-1.0	0.07
AA	36 (52.8)	24 (29.2)	18 (16.7)	0.4	0.2-0.8	0.01
Interaction test [b]						
G×E				0.5	0.4-0.7	7.4×10^{-7}

NW: normal weight (pre-pregnancy BMI: 18.5 – 24.9 kg m^{-2}); OW: overweight (pre-pregnancy BMI: 25.0 – 29.9 kg m^{-2}); OB: obesity (pre-pregnancy BMI ≥30 kg m^{-2}); PTB: preterm birth; IUI: Intra-uterine inflammation; OR: odds ratio; CI: confidence interval.

[a] The pre-pregnancy BMI category-PTB association was analyzed in women carrying CC, CA and AA genotypes at *rs11161721*, separately, using the logistic regression model with adjustment for genotyping batch, genetic ancestry, maternal age at delivery, parity, and infant's gender.

[b] The interaction effect was analyzed in the total sample by adding pre-pregnancy BMI category, *rs11161721* (under the additive genetic model) and their interaction term into the regression model, with adjustment of the same covariates as mentioned above.

Supplementary Table 5. The associations between pre-pregnancy BMI category and overall PTB within each genotype strata at rs11161721, and the rs11161721 × pre-pregnancy BMI interaction effect on overall PTB in the Boston Birth Cohort, after removing those with obesity-related gestational complications

**rs11161721**	**N (%PTB)**			**Model 1[a]**			**Model 2[b]**		
	**NW**	**OW**	**OB**	**OR**	**95%CI**	***P***	**OR**	**95%CI**	***P***
**Mothers without diabetes/gestational diabetes**									
CC	412 (30.6)	272 (42.3)	197 (44.2)	1.3	1.1-1.6	0.001	1.4	1.2-1.7	5.3×10^{-4}
CA	233 (42.9)	125 (44.8)	110 (29.1)	0.8	0.6-1.0	0.03	0.7	0.6-0.9	0.02
AA	40 (57.5)	24 (33.3)	17 (17.6)	0.4	0.2-0.8	0.01	0.4	0.2-0.9	0.02
Interaction test									
G×E				0.6	0.4-0.7	1.1×10^{-6}	0.5	0.4-0.7	6.1×10^{-7}
**Mothers without hypertensive disorders during pregnancy**									
CC	379 (26.4)	251 (35.9)	174 (39.0)	1.3	1.1-1.6	0.005	1.4	1.1-1.7	0.003
CA	210 (38.1)	124 (38.7)	102 (23.5)	0.7	0.6-1.0	0.03	0.7	0.5-0.9	0.01
AA	35 (57.1)	24 (33.3)	17 (17.6)	0.4	0.2-0.8	0.01	0.4	0.2-0.8	0.02
Interaction test									
G×E				0.5	0.4-0.7	2.3×10^{-6}	0.5	0.4-0.7	1.2×10^{-6}
**Mothers without diabetes/gestational diabetes and without hypertensive disorders**									
CC	367 (25.6)	229 (34.5)	150 (37.3)	1.3	1.0-1.6	0.02	1.3	1.1-1.6	0.02
CA	203 (37.9)	110 (39.1)	89 (23.6)	0.7	0.6-1.0	0.03	0.7	0.5-0.9	0.02
AA	34 (55.9)	22 (31.8)	15 (13.3)	0.3	0.1-0.8	0.01	0.3	0.1-0.7	0.01
Interaction test									
G×E				0.5	0.4-0.7	1.2×10^{-5}	0.5	0.4-0.7	8.6×10^{-6}

NW: normal weight (pre-pregnancy BMI: 18.5 – 24.9 kg m^{-2}); OW: overweight (pre-pregnancy BMI: 25.0 – 29.9 kg m^{-2}); OB: obesity (pre-pregnancy BMI ≥30 kg m^{-2}); PTB: preterm birth; TB; term birth; OR: odds ratio; CI: confidence interval.

[a]In model 1, the maternal pre-pregnancy BMI-PTB association was analyzed in women carrying the CC, CA and AA genotype at rs11161721, separately, using the logistic regression model with adjustment for genotyping batch, genetic ancestry, maternal age at delivery, parity, and infant's gender. The interaction effect was analyzed in the total sample by adding the pre-pregnancy BMI category, rs11161721 (under the additive genetic model) and their interaction term into the regression model, with adjustment for the same covariates mentioned above.

[b]In model 2, additional covariates, including smoking during pregnancy, stress during lifetime, stress during pregnancy, and history of illicit drug use, were also adjusted in both the association test and the interaction test.

Supplementary Table 6. The associations between pre-pregnancy BMI category and overall PTB within each genotype strata at rs11161721, and the rs11161721 × pre-pregnancy BMI interaction effect on overall PTB in the Boston Birth Cohort, stratified by infant's gender

**rs11161721**	**N (%PTB)**			**pre-pregnancy BMI category-PTB association [a]**		
	**NW**	**OW**	**OB**	**OR**	**95%CI**	***P*-value**
**Girls**						
CC	208 (32.2)	155 (45.2)	131 (45.8)	1.3	1.1-1.7	0.02
CA	108 (50.0)	64 (43.8)	70 (30.0)	0.7	0.5-1.0	0.03
AA	21 (61.9)	15 (26.7)	9 (11.1)	0.3	0.1-1.5	0.13
G×E [b]				0.5	0.4-0.7	1.7×10^{-5}
**Boys**						
CC	221 (31.2)	153 (45.8)	113 (50.4)	1.6	1.2-2.1	3.2×10^{-4}
CA	136 (39.0)	76 (44.7)	64 (31.3)	0.9	0.6-1.2	0.34
AA	21 (57.1)	11 (45.5)	10 (30.0)	0.5	0.2-1.5	0.24
G×E [b]				0.5	0.4-0.7	1.6×10^{-4}

NW: normal weight (pre-pregnancy BMI: 18.5 – 24.9 kg m^{-2}); OW: overweight (pre-pregnancy BMI: 25.0 – 29.9 kg m^{-2}); OB: obesity (pre-pregnancy BMI ≥30 kg m^{-2}); PTB: preterm birth; OR: odds ratio; CI: confidence interval.

[a]The pre-pregnancy BMI - PTB association was analyzed in women carrying the CC, CA and AA genotype at rs11161721, separately, using the logistic regression model with adjustment for genotyping batch, genetic ancestry, age at delivery, and parity.

[b]The interaction effect was analyzed by adding pre-pregnancy BMI category, rs11161721 (under the additive genetic model) and their interaction term into the logistic regression model, with adjustment for the same covariates as mentioned above.

Supplementary Table 7. The interaction effects between rs11161721 and pre-pregnancy BMI category on overall PTB in the infants from the Boston Birth Cohort and from the GPN study

**rs11161721**[a]	**pre-pregnancy BMI**	**BBC African-American infants**					**GPN African-American infants**				
		**n**	**PTB,%**	**OR**	**95%CI**	**P**[b]	**n**	**PTB,%**	**OR**	**95%CI**	**P**[b]
CC	NW	42	23.8	ref			66	45.5	ref		
CC	OWO	56	35.7	2.0	0.8-5.1	0.13	98	57.1	1.5	0.8-2.8	0.23
CA	NW	20	50.0	3.9	1.3-11.8	0.01	44	43.2	0.8	0.4-1.8	0.63
CA	OWO	28	39.3	2.6	0.9-7.3	0.07	51	49.0	1.1	0.5-2.3	0.84
AA	NW	4[c]	75.0	-	-	-	7	71.4	3.0	0.5-1.7	0.21
AA	OWO	3[c]	66.7	-	-	-	10	20.0	0.3	0.1-1.3	0.10
Interaction test[d]											
G×E				0.4	0.2-1.0	0.05			0.5	0.2-1.1	0.07

BMI: body mass index; NW: normal weight (pre-pregnancy BMI: 18.5 – 24.9 kg m^{-2}); OWO: overweight and/or obesity (pre-pregnancy BMI ≥25kg m^{-2}). BBC: Boston Birth Cohort. PTB: preterm birth; OR: odds ratio; CI: confidence interval.

[a] The rs11161721variant in the infants, which was genotyped in the BBC and imputed in the GPN study.

[b] The analyses were conducted using the logistic regression model, adjusted for the genetic ancestry, parity and infant's gender.

[c] Due to a small sample size in the BBC, children with the rs11161721-AA genotype were combined with their counterparts carrying the rs11161721-AC genotype in the logistic regression analyses.

[d]The interaction effect was analyzed by adding maternal pre-pregnancy BMI category, infant's rs11161721 (under the additive genetic model) and their interaction term into the logistic regression model, with adjustment for the same covariates as mentioned above.

Supplementary Table 8. Analyses of the interaction effects between maternal pre-pregnancy BMI, maternal-origin A allele at rs11161721, and paternal-origin A allele at rs11161721 on overall PTB in 120 African-American mother-infant pairs from the BBC and in 276 African-American mother-infant pairs from the GPN study

rs11161721 A allele [a]	Pre-pregnancy BMI	Mother-infant pairs from the BBC					Mother-infant pairs from the GPN				
		n	PTB, %	OR	95%CI	P^b	n	PTB, %	OR	95%CI	P^b
Maternal-origin A allele											
0	NW	42	21.4	ref			92	43.5	ref		
0	OWO	53	33.9	1.9	0.7-5.1	0.19	124	58.9	1.8	1.0-3.1	0.04
1	NW	14	71.4	8.0	2.0-3.3	0.003	25	56.0	1.6	0.6-4.0	0.31
1	OWO	11	54.5	4.4	1.0-1.9	0.05	35	28.6	0.5	0.2-1.1	0.09
Paternal-origin A allele											
0	NW	46	32.6	ref			84	46.4	ref		
0	OWO	46	37.0	1.4	0.6-3.5	0.48	123	52.0	1.2	0.7-2.1	0.55
1	NW	10	40.0	1.6	0.4-6.8	0.55	33	45.4	0.9	0.4-2.1	0.82
1	OWO	18	38.9	1.2	0.3-3.8	0.82	36	52.8	1.2	0.6-2.7	0.63
**Interaction** [c]											
Maternal-origin A allele × pre-pregnancy OWO				0.3	0.0-2.1	0.21			0.2	0.1-0.6	0.004
Paternal-origin A allele × pre-pregnancy OWO				0.8	0.1-6.3	0.83			1.2	0.4-3.8	0.72

BMI: body mass index; NW: normal weight (pre-pregnancy BMI: 18.5 – 24.9 kg m^{-2}); OWO: overweight (pre-pregnancy BMI ≥25kg m^{-2}); PTB: preterm birth. OR: odds ratio; CI: confidence interval.

[a] The number of maternal-origin A allele or paternal-origin A allele at rs11161721 in the infants.

[b] The analyses were conducted using the logistic regression model, adjusted for maternal genetic ancestry, parity and infant's gender.

[c] The interaction effect was analyzed by adding maternal-origin A allele at rs11161721 (coded as 1 vs 0), paternal-origin A allele at rs11161721, maternal pre-pregnancy BMI category (coded as 0=NW, 1=OWO), and the interaction term between maternal pre-pregnancy BMI category and maternal-origin A allele, as well as the interaction term between maternal pre-pregnancy BMI category and paternal-origin A allele into the same logistic regression model, with adjustment for the same covariates as mentioned above.
